# Cutaneous Fungal Infections in Greek Competitive Swimmers: A Cross-Sectional, Self-Reported Study

**DOI:** 10.3390/jof12030193

**Published:** 2026-03-09

**Authors:** Eleni Sfyri, Niki Tertipi, Vasiliki-Sofia Grech, Vasiliki Kefala, Efstathios Rallis

**Affiliations:** Department of Biomedical Sciences, School of Health and Care Sciences, University of West Attica, GR-12243 Athens, Greece; ntertipi@uniwa.gr (N.T.); vgkrek@uniwa.gr (V.-S.G.); valiakef@uniwa.gr (V.K.); erallis@uniwa.gr (E.R.)

**Keywords:** cutaneous fungal infections, competitive swimmers, tinea pedis, tinea unguium, tinea versicolor

## Abstract

Background/Objectives: Cutaneous fungal infections, specifically tinea pedis, pityriasis versicolor, and tinea unguium (onychomycosis), are common yet under-researched in swimming. This study aimed to evaluate their prevalence and associated risk factors among competitive swimmers in Greece. Methods: An anonymous questionnaire was administered to 1047 participants, comprising parents of minor swimmers and adult athletes. Data collected included demographics, infection history, training characteristics, and hygiene behaviors. Results: The overall prevalence was 16% for tinea pedis, 3.2% for pityriasis versicolor and 3.3% for tinea unguium. Infection rates increased significantly with age and cumulative training years. Behavioral analysis revealed that placing towels and clothes on communal benches was associated with tinea pedis (*p* = 0.031) and pityriasis versicolor (*p* < 0.007). Sharing kickboards correlated with all three infections, while sharing flip-flops was specifically linked to tinea pedis and tinea unguium. Family history was a strong predictor for pityriasis versicolor. Conclusions: This study highlights the high prevalence of fungal skin infections in Greek swimmers, likely due to moisture exposure, shared equipment, and specific hygiene habits.

## 1. Introduction

While swimming is widely regarded as a sport with a low risk of traumatic injury—particularly those related to high-impact contact—the aquatic environment presents distinct dermatological challenges. These range from unique aquagenic conditions to common skin infections [1,2,3]. Consequently, microbial colonization in public swimming facilities remains a critical concern in sports medicine, as pool environments often function as reservoirs for pathogenic microorganisms [4,5,6].

Fungal skin infections are traditionally classified into superficial and cutaneous mycoses based on the depth of tissue invasion and the host immune response. Superficial infections are restricted to the outermost, nonviable layers of the skin, eliciting minimal inflammation, whereas cutaneous infections—predominantly caused by dermatophytes —penetrate keratinized tissues, triggering a marked inflammatory reaction [7,8]. Although this distinction remains clinically relevant for diagnosis and management, the contemporary literature increasingly adopts a unified perspective. Indeed, recent studies frequently address these categories collectively, reflecting the significant clinical overlap and diagnostic complexity inherent in infections involving both dermatophytes and non-dermatophytes [9,10,11]. Epidemiological data indicate that 10–25% of the global population is affected by these infections [12,13]. Such conditions are particularly relevant for athletes training in warm, humid environments that facilitate microbial proliferation [14,15]. Evidence suggests a higher prevalence among elite athletes, driven by risk factors including occlusive microenvironments, hyperhidrosis, and skin micro-trauma. Furthermore, locker rooms and shower facilities act as high-risk transmission zones, potentially compromising athletes’ dermatological health and performance [14,16].

Tinea pedis, commonly known as athlete’s foot, is a cutaneous fungal infection primarily caused by dermatophytes of the Trichophyton genus. While historical data and specific population studies often cite a prevalence of approximately 15% in developed countries, recent global estimates vary significantly, with some reviews suggesting a lower general population prevalence of around 3% [17,18]. Interdigital maceration disrupts the skin barrier, facilitating infection. Since dermatophytes persist on abiotic surfaces, such as tiles and textiles, walking barefoot in communal areas constitutes the primary mode of transmission [1]. In the context of swimming, tinea pedis prevalence has been reported at approximately 8.5%, with males exhibiting higher susceptibility than females [19].

Pityriasis versicolor is a prevalent, often recurrent superficial infection of the stratum corneum caused by lipophilic yeasts of the genus Malassezia. Clinically, it presents as hypopigmented or hyperpigmented scaly macules, predominantly affecting the upper trunk in adults [20,21]. Key predisposing factors include genetic susceptibility, immunosuppression, and ambient humidity. The condition exhibits distinct seasonality with summer exacerbations, while prevalence varies significantly by climate (over 40% in tropical vs. 0.5–1% in temperate regions) [22]. However, high environmental exposure markedly increases rates, reaching 15.5% in fishing communities and 2.1% in sailors [22]. Similarly, athletes show heightened susceptibility, with a prevalence of 12.1% in football players and 9.5% in swimmers [23].

Tinea unguium (onychomycosis), a fungal infection of the nail unit caused predominantly by dermatophytes (70–80%), accounts for approximately 50% of all nail dystrophies [24]. The global prevalence is estimated at 5.5%, falling within the broad range of previously reported estimates (2–8%) [25]. Risk factors include trauma, humidity, and occlusive footwear, making the condition common among swimmers. Beyond aesthetic concerns, tinea unguium can cause pain and discomfort during physical activity [14]. Notably, studies involving recreational swimmers have reported alarming prevalence rates, reaching up to 26% in males, highlighting the high risk associated with aquatic facilities [26].

The aim of this study is to assess the prevalence of cutaneous fungal infections among Greek competitive swimmers. The current literature on this specific demographic is scarce, as the majority of prior research has predominantly focused on the general population, recreational swimmers, or athletes participating in other sporting disciplines [25,26,27].

It is hypothesized that the rigors of competitive swimming, alongside specific hygiene habits and the use of communal equipment, heighten the susceptibility to infections. Currently, the paucity of epidemiological evidence regarding this demographic in Greece constitutes a significant gap in the literature. This research aims to bridge that divide by offering region-specific data, which is essential for establishing targeted, evidence-based preventive measures that safeguard training continuity and athletic performance.

## 2. Materials and Methods

This cross-sectional study investigated the prevalence of cutaneous superficial fungal infections in Greek competitive swimmers. Ethical clearance was granted by the University of West Attica (Ref: 52645, 20 July 2020) and the Hellenic Swimming Federation (Ref: 787, 15 March 2019). Data were collected via an online survey administered between June and December 2021. Due to the logistical difficulties in accessing the total population of competitive swimmers in Greece, a self-selection sampling strategy was deemed the only viable approach.

Recruitment targeted 182 swimming clubs nationwide, resulting in participation from 80 clubs. The data collection process was executed in two distinct phases:

In phase A, the Hellenic Swimming Federation emailed the questionnaire to 143 registered clubs. The remaining 39 clubs, for whom contact details were unavailable, were notified via social media. Respondents in this phase self-reported their club affiliation (Supplementary S1).

In phase B, in order to increase participation, researchers directly contacted coaches and managers of clubs that did not respond in phase A. Sixty clubs were selected for telephone follow-up; swimmers from 33 of these clubs subsequently completed the survey.

**Figure 1:**
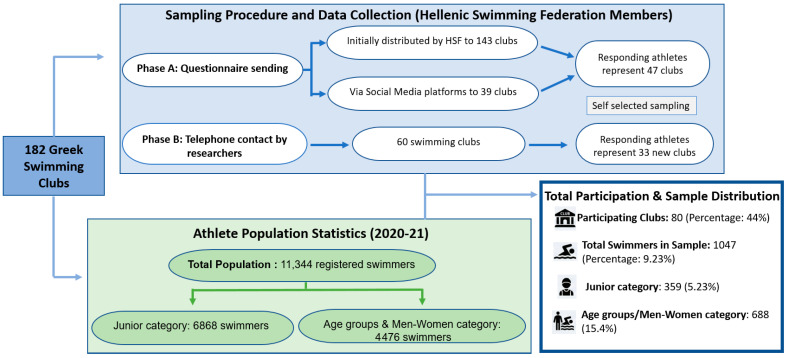
Flowchart of Participant Recruitment and Response.

The target population consisted of 11,344 swimmers (or their parents) registered with the Hellenic Swimming Federation during the 2020–2021 season. A total of 1047 competitive swimmers participated (9.23% of the population), a sample size deemed statistically representative [28]. The cohort included the “Junior category” (ages 9–12), “Age Group categories” (sub-grouped as 13–14, 15–16, and 17–18 years), and the “Men–Women category”. Participation was voluntary and anonymous (Figure 1).

As part of a broader study on dermatological conditions, the questionnaire underwent rigorous validation [28]. Content validity was confirmed by a dermatologist and a methodology expert. The questionnaire consisted of closed-ended questions, categorized as either dichotomous or multiple-choice, with the option for multiple answers. Reliability was assessed via a test–retest procedure involving 57 members of one swimming club over a 15-day interval [29]. Feedback from the initial version regarding question clarity—specifically concerning skin conditions—led to the inclusion of explanatory notes in the final version. Agreement between the two administrations was calculated using Cohen’s Kappa (κ), yielding a value of 0.75. This indicates good reliability, largely attributed to the refinements made based on pilot feedback.

The final questionnaire was administered via Google Forms and comprised two main sections: (1) demographics, training habits, and general skin health, and (2) specific fungal infection details. For the purpose of this specific study, data were extracted regarding demographics, training practices, seasonality and timing of infections. Supplementary Data were collected regarding the training environment (facility type), training load (years of experience and daily duration), and specific pool-related behaviors. The survey also queried family history of fungal infections. To minimize recall bias, participants reporting recurrent infections were instructed to provide details specific to their most recent episode, as this is typically remembered with greater accuracy (Supplementary S1).

### Statistical Analysis

Statistical analysis was performed using IBM SPSS Statistics for Windows, version 26.0 (IBM Corp., Armonk, NY, USA). Categorical variables are reported as frequencies (*n*) and percentages (%). Bivariate associations between categorical variables were assessed using the Chi-square test, while the Chi-square trend test was utilized to examine relationships involving ordinal variables. Bivariate correlations were conducted to identify potential links between cutaneous fungal infections and demographic or training factors (e.g., gender, facility type, years of training, weekly/daily training load) as well as behavioral habits (e.g., walking barefoot, equipment sharing).

Multivariate logistic regression models were employed to evaluate the impact of specific behavioral and equipment-related risk factors on cutaneous fungal infections. Multivariable models assessed behavioral and clinical factors associated with fungal infections. For tinea pedis, exposures included placing clothes or bathrobes on benches and the use of fins, paddles, kickboards, flip-flops, towels, and swimming suits. For pityriasis versicolor, the analyzed variables were placing clothes or bathrobes on benches, kickboard use, and a family history of the infection. For tinea unguium, factors included the use of kickboards, flip-flops, and swimming suits. Associations are reported as odds ratios (ORs) with 95% confidence intervals (CIs). Statistical significance was defined as *p* < 0.05.

## 3. Results

### 3.1. Demographic Characteristics

A total of 1047 swimmers participated in the study (response rate: 9.23%), comprising 577 females (55.1%) and 470 males (44.9%). The majority trained at outdoor facilities (*n* = 637, 60.8%), whereas 470 participants (39.2%) trained in indoor facilities. The most represented age group was 9–12 years (*n* = 359, 34.3%), followed by 13–14 years (*n* = 231, 22.0%), 15–16 years (*n* = 194, 18.6%), 17–18 years (*n* = 112, 10.6%), and ≥18 years (*n* = 151, 14.4%). Training experience was predominantly 7–9 years (*n* = 265, 25.3%) and 4–6 years (*n* = 262, 25.0%). Approximately half of the participants (*n* = 541) reported a daily training duration of two hours, based on responses from both athletes and their parents.

### 3.2. General Characteristics of Cutaneous Fungal Infections and Their Association with Training Routines

#### 3.2.1. Tinea Pedis

A total of 167 swimmers (16%) reported tinea pedis, and almost one-third experienced a single episode. Winter saw the most cases (*n* = 88), followed by lower rates in spring (*n* = 35, 20.5%), summer (*n* = 27, 15.9%), and autumn (*n* = 20, 11.8%). During treatment, 114 swimmers (68.3%) continued their training, whereas 53 suspended it. Medical advice from a dermatologist was reported by 53.9% (*n* = 90), while 44.9% (*n* = 75) handled the condition without medical assistance (Table 1).

Prompted by existing literature, we investigated the association between tinea pedis and tinea unguium. Our analysis revealed that 17.4% (*n* = 24) of swimmers with tinea pedis presented with concomitant tinea unguium. This association was found to be statistically significant, with a remarkably high probability of co-occurrence (OR 3.061; 95% CI: 1.248–7.507; *p* < 0.001).

Bivariate analysis regarding tinea pedis revealed that females demonstrated a higher prevalence (*n* = 102, 17.7%) compared to males (*n* = 65, 13.8%). A statistically significant difference was observed across swimming categories, with adult swimmers exhibiting the highest prevalence of the infection (*n* = 43, 28.5%, *p* < 0.001). Furthermore, long-term swimmers and those who engaged in daily training presented with a higher prevalence of tinea pedis compared to other groups (Table 2).

#### 3.2.2. Pityriasis Versicolor

Pityriasis versicolor was reported by 33 swimmers (3.2%). Most cases (*n* = 24, 72.7%) occurred once, while four swimmers (12.1%) reported it more than six times. The torso (*n* = 14, 53.8%) was the most affected site of the body. More than half of the infections (*n* = 24, 58.5%) occurred in summer. Training continued without interruption for 66.7% (*n* = 22) of the participants, while 33.3% (*n* = 11) stopped training for less than one week. Dermatological consultation and treatment were sought by 81.8% (*n* = 27), and 18.2% (*n* = 6) self-treated (Table 1).

Bivariate analysis revealed no significant difference in prevalence based on gender. However, a statistically significant variation was observed among swimming categories, with adult swimmers exhibiting the highest prevalence (*n* = 14, 9.3%), while the remaining categories showed similar percentages (*p* = 0.01). Additionally, a significant association was found with both the years of training (*p* < 0.001) and the duration of daily training. Specifically, swimmers training up to 2 h daily demonstrated the highest infection rate (*n* = 25, 4.6%, *p* < 0.001) (Table 2).

#### 3.2.3. Tinea Unguium

Tinea Unguium was reported by 35 swimmers (3.3%). In 57.1% (*n* = 20) of cases, the condition occurred only once. The infection was reported by nearly half of the participants in the winter season. The majority (*n* = 21, 60%) continued training, while 74.3% (*n* = 26) consulted a dermatologist and received treatment. A smaller proportion (*n* = 9, 25.7%) self-managed the infection (Table 1).

Bivariate analysis indicated that females exhibited a higher prevalence (*n* = 23, 4%) compared to males (*n* = 12, 2.6%). A statistically significant difference was observed among swimming categories, with adult swimmers demonstrating the highest prevalence of tinea unguium (*n* = 21, 13.9%, *p* = 0.001), a finding consistent with their long-term engagement in the sport (*p* < 0.001). Furthermore, the type of swimming facility was found to be a significant factor; participants utilizing indoor swimming pools exhibited higher infection rates (*n* = 20, 4.7%, *p* = 0.027) (Table 2).

### 3.3. Correlation Between Swimmers’ Behavior and Habits and the Development of Cutaneous Fungal Infections

Statistical analysis was performed to investigate the relationship between behavioral factors in the pool area and cutaneous fungal infections. Tinea pedis showed a significant association with the sharing of swimming equipment, specifically fins (*p* = 0.05), paddles (*p* < 0.001), kickboards (*p* = 0.043), flip-flops (*p* < 0.001), towels or bathrobes (*p* < 0.001), and swimming suits (*p* = 0.002). Subsequent analysis indicated that sharing flip-flops (OR 2.081, 95% CI: 1.398–3.098, *p* < 0.001), towel/bathrobe (OR 2.484, 95% CI: 1.542-4.004 *p* < 0.001) and puddles (OR 1.447, 95% CI: 1.004–2.087, *p* = 0.048) increased the risk of tinea pedis (Table 3).

Regarding pityriasis versicolor, 29 swimmers (4%) reported placing personal clothing on pool benches. This practice was associated with a fourfold increase in the risk of infection (OR 4.034, 95% CI: 1.404–11.596, *p* = 0.007). Additionally, sharing kickboards and having a family history of the infection were significant risk factors. Specifically, the odds of developing pityriasis versicolor doubled for those sharing kickboards (OR 2.497, 95% CI: 1.033–6.037, *p* = 0.05) and tripled for individuals with a family history of the condition (OR 3.537, 95% CI: 1.641–7.625, *p* = 0.001).

Behaviors reported by the 33 subjects affected by tinea unguium included walking barefoot on pool decks (*n* = 25, 3.3%) and placing bathrobes or clothes on benches (*n* = 28, 4.1%). Statistical analysis further identified that sharing kickboards (*p* = 0.049), flip-flops (*p* = 0.05), and swimming suits (*p* = 0.003) were significant factors associated with the infection (Table 3).

## 4. Discussion

Swimming pools constitute a specific environment where high humidity and temperature favor the growth and transmission of pathogenic fungi [30,31]. Consequently, cutaneous fungal infections are frequently observed among swimmers, often with higher prevalence rates compared to the general population. While the presence of dermatophytes on pool surfaces and decks is well-documented [32], the specific behavioral factors contributing to transmission among competitive athletes remain under-explored. In this study, we aimed to investigate the prevalence of these infections in competitive swimmers and to evaluate the role of hygiene habits and equipment sharing in the spread of the disease.

Our data revealed a 16% prevalence of tinea pedis among competitive swimmers, falling within the 13.2–22.2% range observed in students attending swimming lessons [1,33]. Notably, this rate is substantially higher than the estimated 3% prevalence reported in the general global population [18]. To the best of our knowledge, this is the first study to specifically investigate the prevalence of fungal infections within the competitive swimming population.

We observed a female predilection (17.7%), which stands in contrast to general population data where prevalence is typically higher in males than females [34]. Similarly, other population-based study has reported male-to-female prevalence ratios ranging from 2:1 to 3:1, attributed to behavioral factors such as occlusive training shoes and higher exposure in communal facilities [35]. Furthermore, susceptibility appeared to be a function of cumulative exposure; age and systematic training program were positively associated with infection rates, which escalated from 11.6% in adolescents to 28.5% in older groups [18]. Similarly, the prevalence in our pre-competitive pediatric cohort (6.1%) exceeded the 4% baseline reported for the general pediatric population [36,37], though it is worth noting that pediatric rates exhibit significant heterogeneity across studies [10].

A notable finding was the link between shared equipment and infection [10,37]. These findings corroborate reports of fungal contamination in pool water and surfaces [32], a risk exemplified by the high prevalence of mixed infections (46%) observed in pool employees [38]. Crucially, our study identified a strong statistical correlation between tinea pedis and the sharing of equipment—specifically fins, paddles, kickboards, and towels [39]. These items serve as fomite vectors, facilitating transmission through direct contact with plantar and palmar surfaces. Data analysis revealed a significant correlation between the infection and the concomitant presence of tinea unguium [26,40].

The observed 3.2% prevalence of pityriasis versicolor exceeds the 0.8–1.1% range typically reported for general populations in temperate climates yet remains substantially lower than the 28.5% seen in tropical regions [21,22]. Our findings align with the 3.8% prevalence observed in recreational pool users [41] yet contrast with the 9.5% reported by Aytimur et al. [23] in a small cohort of swimmers. Thus, the warm and humid environment of swimming facilities appears to be a key factor in disease development, further exacerbated by the environmental conditions in Greece, particularly during warmer seasons.

While Pityriasis versicolor affected both sexes, age emerged as a key determinant, peaking in adolescents and young adults. This predilection is attributed to maximal sebaceous activity, which provides a lipid-rich substrate for yeast proliferation—a susceptibility likely compounded by cumulative exposure to the aquatic environment [13,22,42]. It is worth noting that a recent study by Amsri et al. underscores the role of amoebae as environmental reservoirs guiding yeast evolution via selective pressure. This interaction represents a pivotal adaptive mechanism with profound clinical implications. The proposed theoretical model depicts these complex interactions, providing a crucial framework for further research [43].

Of particular significance was the robust statistical correlation between truncal lesions and the use of shared swimming equipment, specifically kickboards. A similar risk was noted with placing garments on communal benches [13,44]. To our knowledge, the specific implication of kickboards has not been previously documented, highlighting a potential novel fomite transmission route. In contrast, the strong association observed with a positive family history aligns with established data suggesting a genetic susceptibility to the infection [45]. The clinical appearance of the lesions drove health-seeking behavior in the majority of participants. Regarding prevention, while earlier protocols recommended the temporary exclusion of infected swimmers and rigorous facility disinfection [41], such strict containment measures are rarely implemented in modern practice.

The prevalence of tinea unguium in our study was recorded at 3.3%. This finding closely corresponds to population-based data from Europe and North America, which place the general population rate at approximately 4.3% [46]. Broader reports, in the same regions, show rates ranging from 8.7% to 24% [47]. This contrasts sharply with the 40% prevalence reported in recreational swimmers, a discrepancy likely attributable to that cohort’s older age profile (>30 years) and non-competitive status [26]. Studies specifically investigating dermatomycoses in competitive-level swimmers were not identified in the existing literature. Although female swimmers exhibited a slightly higher rate (4%) than males (2.6%), the difference was not statistically significant. Our finding aligns with recent large-scale epidemiological data, such as the ‘All of Us’ database study, which reported a 54.2% female majority [48]. This contrasts with literature often reporting a male predominance [42], though some studies attribute rising rates in women to occlusive footwear, trauma, increased athletic participation and cosmetic procedures [49,50].

Athlete age appeared to be associated with the manifestation of the disease, as well as with the duration (years and hours) of training. Infection was less common among children, a finding likely attributable to lower exposure to contaminated environments and faster nail growth rates [51]. Toenails were the predominant site of infection, a finding likely attributable to their slower growth dynamics and direct, repeated contact with fungal reservoirs on pool decks and locker room floors [32,52]. Furthermore, it was observed that sharing swimming suits may contribute to the transmission of the infection. Notably, indoor facilities were associated with a higher prevalence of onychomycosis (4.9%) compared to outdoor facilities (2.4%), likely due to higher environmental humidity. Given the requirement for prolonged therapeutic regimens, the majority of affected participants sought specialist dermatological care; notably, however, this did not result in training discontinuation.

Although water quality parameters were not assessed in this study, previous research highlights their critical role in fungal ecology. Physical-chemical factors, particularly residual chlorine levels and pH, are known to directly influence fungal survival and proliferation in swimming pools [30,31]. Consequently, inadequate water maintenance could be a contributing factor to the prevalence rates observed in our study.

The present study has certain limitations. The cross-sectional design precludes establishing causality, while self-reporting may introduce recall bias. However, selection bias was mitigated by the robust sample size targeting the entire population. A limitation of this study is the reliance on self-reported medical history without independent microbiological confirmation. Although participants were asked to report infections most diagnosed by a dermatologist, we could not verify whether confirmatory testing was performed in every case, as recommended by current guidelines. Nevertheless, the validity of the data is supported by the fact that the reported cases were based on prior specialist assessment and prescribed antifungal treatment [53,54]. Additionally, environmental determinants, such as pool water physicochemical quality, were not evaluated. Since improper chlorination or pH levels can facilitate fungal survival [30], future research should incorporate these variables for a holistic risk assessment. Finally, as the study focused on competitive swimmers in a warm climate, findings may not be fully generalizable to recreational users or colder geographic regions.

This study elucidates the epidemiology of superficial fungal infections in competitive swimmers, underscoring the critical role of shared fomites in transmission. These findings necessitate revised preventive strategies [55]. Crucially, as training often continues during infection, educational interventions emphasizing hygiene, protective footwear, and the avoidance of shared equipment are paramount [26,55,56]. Concurrently, rigorous facility disinfection is essential to mitigate environmental fungal loads [41,57]. Future research should quantify environmental contamination to establish safer training conditions and clear ‘return-to-play’ guidelines.

## 5. Conclusions

The data presented herein hold particular relevance for competitive swimmers in Greece. The notable prevalence of these three superficial fungal infections points to a specific risk profile for this group, driven by the aquatic setting and hygiene practices related to communal equipment. Mitigating the impact of these infections necessitates a shift towards rigorous preventive measures, increased awareness, and timely medical intervention to protect the well-being of the swimming population.

## Figures and Tables

**Table 1:** Cutaneous fungal infections (Tinea Pedis–Pityriasis versicolor–Tinea Unguium).

	Tinea Pedis *n* (%)	Pityriasis Versicolor *n* (%)	Tinea Unguium (%)
Yes	167 (16)	33 (3.2)	35 (3.3)
No	880 (84)	1014 (96.8)	1012 (96.7)
Number of reported infection episodes			
One	63 (37.6)	24 (72.7)	20 (57.1)
Two	27 (16.2)	2 (6.1)	5 (14.3)
Three	33 (19.8)	3 (9.1)	2 (5.7)
Four	13 (7.8)	0	0
Five	8 (4.8)	0	2 (5.7)
≥Six	23 (13.8)	4 (12.1)	6 (17.2)
Infection sites			
Face	0	4 (15.4)	0
Torso	0	14 (53.8)	0
Upper limbs	0	4 (15.4)	5 (14.2)
Lower limbs	167 (100)	4 (15.4)	30 (85.7)
Season during which infection occurred			
Winter	88 (51.8)	2 (4.9)	20 (47.7)
Spring	35 (20.5)	7 (17.1)	6 (14.3)
Summer	27 (15.9)	24 (58.5)	8 (19)
Autumn	20 (11.8)	8 (19.5)	8 (19)
Duration of training interruption due to infection			
<1 week	47 (28.1)	11 (33.3)	8 (22.9)
<1 month	6 (3.6)	0	4 (11.4)
>1 month	0	0	2 (5.7)
Non-training interruption	114 (68.3)	22 (66.7)	21 (60)
Type of diagnosis and treatment received			
Dermatologist consultation and treatment	90 (53.9)	27 (81.8)	26 (74.3)
Only pharmaceutical treatment	75 (44.9)	6 (18.2)	9 (25.7)
None of the above	2 (1.2)	0	0

**Table 2:** Bivariate analysis using Tinea Pedis, Pityriasis Versicolor, and Tinea Unguium as dependent variables.

	Tinea Pedis (*n*%)			Pityriasis Versicolor (*n*%)			Tinea Unguium (*n*%)		
Characteristics	Yes	No	*p* Value	Yes	No	*p* Value	Yes	No	*p* Value
Gender			*p* = 0.091 ^a^			*p* = 0.772 ^a^			*p* = 0.200 ^a^
Male	65 (13.8)	405 (86.2)		14 (3)	456 (97)		12 (2.6)	458 (97.4)	
Female	102 (17.7)	475 (82.3)		19 (3.3)	577 (96.7)		23 (4)	554 (96)	
Swimming age categories			*p* < 0.001 ^b^			*p* = 0.010 ^b^			*p* < 0.001 ^b^
Junior									
9–12 years old	22 (6.2)	337 (93.8)		5 (1.4)	354 (98.6)		5 (1.4)	354 (98.6)	
Age groups									
13–14 years old	30 (13)	201 (87)		6 (2.6)	125 (97.4)		3 (1.3)	228 (98.7)	
15–16 years old	38 (19.6)	156 (80.4)		4 (2.1)	190 (97.9)		4 (2.1)	190 (97.9)	
17–18 years old	24 (21.4)	88 (78.6)		4 (3.6)	108 (96.4)		2 (1.8)	110 (98.2)	
>18 years old	43 (28.5)	108 (71.5)		14 (9.3)	137 (90.7)		21 (13.9)	130 (86.1)	
Type of swimming facility			*p* = 0.809 ^a^			*p* = 0.738 ^a^			*p* = 0.027 ^a^
Outdoor facility	103 (16.2)	534 (83.8)		21 (3.3)	616 (96.7)		15 (2.4)	622 (97.6)	
Indoor facility	64 (15.6)	346 (84.4)		12 (2.9)	398 (97.1)		20 (4.9)	390 (95.1)	
Years of training			*p* < 0.001 ^b^			*p* < 0.001 ^b^			*p* < 0.001 ^b^
≤3 years	9 (9)	91 (91)		1 (1)	99 (99)		1 (1)	99 (99)	
4–6 years	17 (6.5)	245 (93.5)		4 (1.5)	258 (98.5)		4 (1.5)	258 (98.5)	
7–9 years	42 (15.8)	223 (84.2)		5 (1.9)	260 (98.1)		5 (1.9)	260 (98.1)	
10–12 years	37 (17.1)	179 (82.9)		4 (1.9)	212 (98.1)		3 (1.4)	213 (98.6)	
≥12 years	62 (30.4)	142 (69.6)		19 (9.3)	185 (90.7)		22 (10.8)	182 (89.2)	
Weekly training frequency			*p* = 0.062 ^b^			*p* = 0.537 ^b^			*p* = 0.129 ^b^
≤3 times	23 (15.4)	126 (84.6)		5 (3.4)	144 (96.6)		9 (6)	140 (94)	
4–5 times	37 (12.1)	270 (87.9)		7 (2.3)	300 (97.9)		10 (3.3)	297 (96.7)	
≥6 times	107 (18.1)	484 (81.9)		20 (3.4)	571 (96.6)		16 (2.7)	575 (97.3)	
Daily training duration			*p* = 0.005 ^b^			*p* = 0.017 ^b^			*p* = 0.069 ^b^
≤1.5 h/day	32 (10.3)	278 (89.7)		6 (1.9)	304 (98.1)		9 (2.9)	301 (97.1)	
2 h/day	101 (18.6)	442 (81.4)		25 (4.6)	518 (95.4)		24 (4.4)	519 (95.6)	
>2 h/day	34 (17.5)	160 (82.5)		2 (1)	192 (99)		2 (1)	192 (99)	

Values are expressed as *n* (%), unless stated otherwise. ^a^ X^2^ test; ^b^ X^2^ test for trend.

**Table 3:** Correlation between cutaneous fungal infections and swimmers’ behavior and habits.

	Tinea Pedis (*n*%)		Pytiriasis Versicolor (*n*%)		Tinea Unguium (*n*%)	
	Yes	No	Yes	No	Yes	No
Walking barefoot on the pool deck						
	*p* = 0.134		*p* = 0.715		*p* = 0.886	
Yes	129 (17)	630 (83)	23 (3)	736 (97)	25 (3.3)	734 (967)
No	38 (13.2)	250 (86.8)	10 (3.5)	278 (96.5)	10 (3.5)	278 (96.5)
Placing bathrobes or clothing on the pool bench						
	*p* = 0.031		*p* = 0.007		*p* = 0.067	
Yes	122 (17.7)	567 (82.3)	29 (4.2)	660 (95.8)	28 (4.1)	658 (95.9)
No	45 (12.6)	313 (87.4)	4 (1.1)	354 (98.9)	7 (1.9)	354 (98.1)
Sharing swimming equipment						
Fins	*p* < 0.05		*p* = 0.185		*p* < 0.359	
Yes	75 (18.7)	326 (81.3)	9 (2.2)	392 (97.8)	16 (4)	385 (96)
No	92 (14.2)	554 (85.8)	24 (3.7)	622 (96.3)	19 (2.9)	627 (97.1)
Paddles	*p* < 0.001		*p* = 0.265		*p* = 0.062	
Yes	86 (21)	324 (79)	16 (3.9)	394 (96.1)	19 (4.6)	391 (95.4)
No	81 (12.7)	556 (87.3)	17 (2.7)	620 (97.3)	16 (2.5)	621 (97.5)
Kickboard	*p* = 0.043		*p* = 0.050		*p* = 0.049	
Yes	124 (17.5)	583 (82.5)	27(3.8)	680 (96.2)	29 (4.1)	678 (95.9)
No	43 (12.6)	297 (87.4)	7 (2.1)	333 (97.9)	6 (1.8)	334 (98.2)
Flip-flops	*p* < 0.001		*p* = 0.774		*p* = 0.05	
Yes	58 (27.5)	153 (72.5)	6 (2.8)	205 (97.2)	11 (5.2)	200 (94.8)
No	109 (13)	727 (87)	27 (3.2)	809 (96.8)	24 (2.9)	812 (97.1)
Towel/bathrobes	*p* < 0.001		*p* = 0.551		*p* = 0.492	
Yes	28 (29.8)	66 (70.2)	2 (2.1)	92 (97.9)	2 (2.1)	92 (97.9)
No	139 (14.6)	814 (85.4)	31 (3.3)	922 (96.7)	33 (3.5)	920 (96.5)
Swimming suits	*p* = 0.002		*p* = 0.945		*p* = 0.003	
Yes	33 (25.2)	989 (74.8)	4 (3.1)	127 (96.9)	10 (7.6)	121 (92.4)
No	134 (14.6)	782 (85.4)	29 (3.2)	887 (96.8)	25 (2.7)	891 (97.3)
Family history of fungal infection	*p* = 0.937		*p* = 0.001		*p* = 0.271	
Yes	19 (15.7)	102 (84.3)	10 (8.3)	111 (91.7)	2 (1.7)	119 (98.3)
No	148 (16)	778 (84)	23 (2.5)	903 (97.5)	33 (3.6)	893 (96.4)

## Data Availability

Data are contained within the article.

## Supplementary Materials

The following supporting information can be downloaded at: https://www.mdpi.com/article/10.3390/jof12030193/s1, Supplementary S1: Questionnaire.
