# Supplementary material for: Cutaneous Fungal Infections in Greek Competitive Swimmers: A Cross-Sectional, Self-Reported Study
Source: J Fungi (Basel). 2026 Mar 9;12(3):193. doi: 10.3390/jof12030193 (PMC13027808; doi:10.3390/jof12030193)
Supplement: Supplementary file 1 [file jof-12-00193-s001.zip › jof-4084477-supplementary.pdf]

VOLUNTEER CONSENT FORM FOR PARTICIPATION IN RESEARCH

Coordinating Institution: University of West Attica, Department of Biomedical Sciences

Ethics Approval: Ethics Committee by University of West Attica (52645-20 July 2020)

Approved by the Hellenic Swimming Federation (787/15-03-2019)

Title: Epidemiological Study of Dermatological Diseases in Competitive Swimmers

Purpose of the Study:

This research aims to examine the prevalence of skin conditions among competitive swimmers. This nationwide study represents a groundbreaking initiative in documenting dermatological issues faced by these athletes. It will furnish the Hellenic Swimming Federation (KOE), which includes its teams and athletes, with crucial insights into swimmer safety concerning cutaneous infectious diseases as well as environmental factors such as water quality, temperature, sunlight, and wind. The findings will help identify the most prevalent skin conditions among Greek swimmers, investigate their potential causes, and recommend suitable protective strategies.

The study focuses on swimmers aged 9 to 12 years old, age group categories (+13 years old) and adult swimmers.

Main Investigator's Full Name: Eleni Sfyri, Lecturer

Contact Information: Email: elsfiri@uniwa.gr

Consent:

I provide my consent to participate in this research study, either for myself or on behalf of my child.

I acknowledge that the data collected may be utilized in conference presentations and/or published in scientific journals.

Participation is voluntary, and I am not obligated to answer every question; I also have the right to withdraw from the study at any time, even after signing this consent form.

The anonymity of my participation or that of my child will be preserved.

I have reviewed the information provided above and consent to participate in the study, either personally or on behalf of my child.

NOTE

We invite you to participate in a research about cutaneous infections in Greek Competitive Swimmers. This research is carried out with the approval of the Swimming Federation of Greece (documents 787/e – 15/3/2019, 2275/e - 22/6/2020) and in the framework of its cooperation with the University of West Attica / School of Health and Welfare Sciences / Department of Biomedical Sciences. The Ethics committee of research of the University of West Attica has, also, approved this research (no. 48855/8.7.2020).

The research concerns swimmer's junior category, 9 to 12 years old, age group categories (13-14, 15-16, 17-18) and Men Women. The questionnaire will be completed by parents/guardians if the swimmers is underage.

- Participation is strictly anonymous.
- In case you have more than one child - swimmer in these categories please repeat the procedure.
- Completing the questionnaire takes about 15 minutes.

The research is headed by Mrs. Sfyri Eleni, Lecturer in Applications - Department of Biomedical Sciences, University of West Attica, Bachelor in Physical Education/Swimming.
E mail address: elsfiri@uniwa.gr

You must consent if you want to participate to this study.

Consent ☐ No consent ☐

Part A

In Part A you note with an X your answer in the last column where required.

Symbol * means that the answer is mandatory

1.	*Gender	Male	
		Female	
2.	*Swimmer's age category	9 – 12 years old	
		13 – 14 years old	
		15 – 16 years old	
		17 – 18 years old	
		Older than 18 years old	
3.	Swimming club (optionally)	…………………………………………………………	
5.	*Type of swimming pool facility	Outdoor facility	
		Indoor facility	
6.	*Years of training	Less/equal than 3 years	
		4 – 6 years	
		7 – 9 years	
		10 – 12 years	
		More than 12 years	
7.	*Weekly training frequency	Less/equal than 3 times	
		4 – 5 times	
		More than 6 times	
8.	*Daily training duration	Less/equal than 1.5 hours/day	
		2 hours/day	
		More than 2 hours/day	
9.	*Behavior in the swimming pool facility (more than one options):	Walking barefoot on the pool's deck	
		Placing clothes/bathrobes on the pool's bench	
10.	*Sharing equipment (more than one options)	Training fins	
		Hand paddles	
		Kickboard	
		Flip flops	
		Towel/bathrobe	
		Swimming suit	
12.	*History of skin manifestations before taking up swimming	Yes	
		No	
13.	Skin manifestations occurring during years of swimming training	Yes	
		No	

PART B.

In this part of the questionnaire, answer the questions about the following skin infections you have experienced as many years as you swim.

- in C, D, E, F, G questions report only on the last time of infections.

**A. CUTANEOUS MANIFESTATIONS**		
Fungal infections	Explanation	Yes
Tinea pedis	Tinea pedis, widely known as athlete's foot, is a common and contagious fungal skin infection that usually begins between the toes and causes intense itching, stinging, and peeling.	
Pityriasis versicolor	Pityriasis versicolor is a common and harmless fungal skin infection that causes small, scaly patches of discoloration—either lighter or darker than your surrounding skin—typically appearing on the back, chest, or shoulders.	
Tinea Unguium (Onychomycosis)	Tinea unguium, commonly known as fungal nail infection, is a persistent infection that typically causes the nails—most often on the toes—to become discolored, thickened, and brittle	

SKIN INFECTIONS	B. NUMBER OF INFECTIOUS EVENTS*					
	One	Two	Three	four	Five	≥six
Tinea pedis						
Pityriasis versicolor						
Tinea Unguium (Onychomycosis)						

	C. SITES OF INFECTIONS (more than an answer) *				
	Face	Torso	Upper limbs	Lower limbs	Soles
Tinea pedis					
Pityriasis versicolor					
Tinea Unguium (Onychomycosis)					

	D. INTERRUPTION PERIOD OF THE TRAINING PROGRAM DURING TREATMENT OF THE SKIN INFECTION*			
	No training interruption	Less than a week	Less than a month	More than a month
Tinea pedis				
Pityriasis versicolor				
Tinea Unguium (Onychomycosis)				

	E. SEASON OF SKIN INFECTIONS (more than an answer)*			
	Winter	Spring	Summer	Autumn
Tinea pedis				
Pityriasis versicolor				
Tinea Unguium (Onychomycosis)				

	F. DERMATOLOGIST EXAMINATION FOR CERTAIN SKIN INFECTIONS *		G. TREATMENT FOR CERTAIN SKIN INFECTIONS*	
	Yes	No	Yes	No
Tinea pedis				
Pityriasis versicolor				
Tinea Unguium (Onychomycosis)				

	FAMILY HISTORY OF CUTANEOUS FUNGAL INFECTIONS	
	Yes	No
Tinea pedis		
Pityriasis versicolor		
Tinea Unguium (Onychomycosis)		
